# Transcriptomics of receptive endometrium in women with sonographic features of adenomyosis

**DOI:** 10.1186/s12958-021-00871-5

**Published:** 2022-01-03

**Authors:** Erika Prašnikar, Tanja Kunej, Mario Gorenjak, Uroš Potočnik, Borut Kovačič, Jure Knez

**Affiliations:** 1 Department of Reproductive Medicine and Gynaecological Endocrinology, University Medical Centre Maribor, 2000 Maribor, Slovenia; 2 Department of Animal Science, Biotechnical Faculty, University of Ljubljana, 1230 Domžale, Slovenia; 3 Centre for Human Molecular Genetics and Pharmacogenomics, Faculty of Medicine, University of Maribor, 2000 Maribor, Slovenia; 4 Laboratory for Biochemistry, Molecular Biology and Genomics, Faculty of Chemistry and Chemical Engineering, University of Maribor, 2000 Maribor, Slovenia; 5 Department of Gynaecology, University Medical Centre Maribor, 2000 Maribor, Slovenia

**Keywords:** Adenomyosis, Assisted reproductive techniques (ART), Data integration, Endometrial receptivity, Enrichment pathway analysis, Omics approaches, RNA-seq, Systems biology, Transcriptomics, Window of implantation

## Abstract

**Background:**

Women with uterine adenomyosis seeking assisted reproduction have been associated with compromised endometrial receptivity to embryo implantation. To understand the mechanisms involved in this process, we aimed to compare endometrial transcriptome profiles during the window of implantation (WOI) between women with and without adenomyosis.

**Methods:**

We obtained endometrial biopsies LH-timed to the WOI from women with sonographic features of adenomyosis (n=10) and controls (n=10). Isolated RNA samples were subjected to RNA sequencing (RNA-seq) by the Illumina NovaSeq 6000 platform and endometrial receptivity classification with a molecular tool for menstrual cycle phase dating (beREADY®, CCHT). The program language R and Bioconductor packages were applied to analyse RNA-seq data in the setting of the result of accurate endometrial dating. To suggest robust candidate pathways, the identified differentially expressed genes (DEGs) associated with the adenomyosis group in the receptive phase were further integrated with 151, 173 and 42 extracted genes from published studies that were related to endometrial receptivity in healthy uterus, endometriosis and adenomyosis, respectively. Enrichment analyses were performed using Cytoscape ClueGO and CluePedia apps.

**Results:**

Out of 20 endometrial samples, 2 were dated to the early receptive phase, 13 to the receptive phase and 5 to the late receptive phase. Comparison of the transcriptomics data from all 20 samples provided 909 DEGs (p<0.05; nonsignificant after adjusted p value) in the adenomyosis group but only 4 enriched pathways (Bonferroni p value < 0.05). The analysis of 13 samples only dated to the receptive phase provided suggestive 382 DEGs (p<0.05; nonsignificant after adjusted p value) in the adenomyosis group, leading to 33 enriched pathways (Bonferroni p value < 0.05). These included pathways were already associated with endometrial biology, such as “Expression of interferon (IFN)-induced genes” and “Response to IFN-alpha”. Data integration revealed pathways indicating a unique effect of adenomyosis on endometrial molecular organization (e.g., “Expression of IFN-induced genes”) and its interference with endometrial receptivity establishment (e.g., “Extracellular matrix organization” and “Tumour necrosis factor production”).

**Conclusions:**

Accurate endometrial dating and RNA-seq analysis resulted in the identification of altered response to IFN signalling as the most promising candidate of impaired uterine receptivity in adenomyosis.

**Supplementary Information:**

The online version contains supplementary material available at 10.1186/s12958-021-00871-5.

## Executive summary of the study

1. Adenomyosis has been associated with lower pregnancy rate in infertility treatments.
2. Molecular knowledge of endometrial receptivity in women with adenomyosis is scarce and limited to studies with selected candidate genes and one genome-wide study performed using microarrays.
3. Therefore, we performed the first transcriptome sequencing of endometrial samples LH-timed to the expected WOI (LH+7 – LH+9) between women with (n = 10) and without (n = 10) sonographic features of adenomyosis.
4. Transcriptomics data comparison of 10 adenomyosis cases and 10 normal controls provided 909 DEGs (p<0.05; nonsignificant after adjusted p value), but functional enrichment analysis identified 4 pathways (Bonferroni p value < 0.05) that were not directly associated with endometrial biology.
5. Retrieved endometrial biopsies were applied for the external molecular tool beREADY® (CCHT, Estonia) to verify their receptivity status on the basis of the gene expression signature associated with endometrial receptivity. Out of 20 samples, 2 were classified as early receptive, 13 as receptive and 5 as late receptive.
6. Two early- and 5 late-receptive samples were excluded from the RNA-seq dataset to prevent the impact of early- and late-secretory phases of the menstrual cycle on transcriptomics analysis associated with endometrial receptivity. The RNA-seq dataset of the remaining 8 adenomyosis cases and 5 control receptive endometrial samples was reanalysed, and 382 DEGs (p < 0.05; nonsignificant after adjusted p value) were identified, resulting in 33 enriched pathways (Bonferroni p value < 0.05) that have already been associated with endometrial biology.
7. The 382 identified DEGs were further integrated with the most extensive set of genes from the literature associated with endometrial receptivity in the healthy uterus, endometriosis (model disease to study persistence of gynaecological pathology on endometrial molecular organization) and adenomyosis to provide candidate pathways characterizing the role of adenomyosis on endometrial molecular organization.
8. Integrative enrichment analysis provided candidate pathways that may indicate a unique effect of adenomyosis on endometrial molecular organization (e.g., “Expression of IFN-induced genes”) and its interference with endometrial receptivity establishment (e.g., “Extracellular matrix organization”, “Tumour necrosis factor production” and “Regulation of reproductive process”).
9. Identification of robust endometrial pathways and associated genes could lead to the development of molecular tools for endometrial receptivity examination that would be specific for women with adenomyosis.
10. Accurate endometrial receptivity examination in infertile adenomyosis patients could better verify whether endometrial-associated factors are a source of recurrent implantation failures.

## Background

Adenomyosis is a common acquired uterine anomaly characterized by the presence of endometrial glands and stroma within the myometrium. Advances in imaging techniques in the last decade have enabled the diagnosis of adenomyosis [1] in a large proportion of women undergoing infertility diagnostics [2, 3]. Since subtle sonographic signs of adenomyosis are becoming easier to recognize, adenomyosis is diagnosed with increasing frequency. Previous retrospective studies have shown the association between adenomyosis and lower embryo implantation rates and higher miscarriage rates [4–6].

Several functional and molecular aberrations could be responsible for altered endometrial receptivity to embryo implantation and lower fecundity in women with adenomyosis. It has been suggested that the disruption of the junctional zone architecture by adenomyosis could lead to altered contractility and interrupt endometrial receptivity [7, 8]. Other suggested causes affecting endometrial receptivity in women with adenomyosis could be increased levels of oxidative stress [9–11], abnormal endometrial vascularity [12, 13] and functional disorganization at the molecular level [14–17].

In our previous study [18], we gathered proteins, genes and functional noncoding RNAs (ncRNAs) shown to be dysregulated in the endometrium of women with adenomyosis during the expected window of implantation (WOI). Bioinformatics approaches were used to integrate retrieved loci with endometrial receptivity genes from the literature associated with healthy (normal) uteri to identify candidate dysregulated mechanisms involved in the regulation of embryo implantation in adenomyosis. In addition, we included better characterized endometriosis as a model disorder to study the impact of gynaecological pathology on endometrial molecular organization [18]. Numerous published genome-wide studies associated with the endometrial molecular background in women with endometriosis enabled us to develop a catalogue of genes sorted according to the phases of the menstrual cycle [19]. Genes sorted in the mid-secretory phase corresponding to the appearance of the WOI were used for the integrative analysis mentioned above [18]. The identified enriched “Signalling by interleukins” and “Interleukin-4 and interleukin-13 signalling” pathways were prioritized, and the corresponding mapped *LIF*, *SOCS3*, *IL10*, *IL6*, *JUNB* and *FOS* genes were validated. Since downregulated expression levels of selected genes in adenomyosis compared to the control group showed no statistical significance, we assumed that comprehensive endometrial transcriptomics profiling would be an appropriate next step to identify adenomyosis-specific loci [18].

To date, there is only one transcriptomics study [20] profiling the endometrium in the expected WOI using microarrays, which identified 34 differentially expressed genes (DEGs) in women with adenomyosis wishing to conceive compared to healthy women [20]. The methodological improvement of transcriptome profiling from hybridization-based microarrays to next-generation sequencing (NGS) platforms provides more comprehensive insight into expression signatures and enables identification of minor differences between study groups [21]. Millions of reads generated by RNA sequencing (RNA-seq) can be aligned to a reference genome, reference transcripts or references assembled de novo for the entire transcriptome to be surveyed. Thus, additional biological constituents can be identified, and a more precise assessment of transcript expression levels can be obtained [22].

The first aim of this study was to perform RNA-seq of endometrial samples dated to the WOI between women with and without sonographic features of adenomyosis to identify DEGs. The second aim was to perform enrichment analysis of identified DEGs alone and together with endometrial receptivity genes from the literature to provide robust candidate pathways related to altered molecular background of endometrial receptivity in adenomyosis.

## Methods

### Study cohorts

We designed a prospective observational study including women scheduled for medically assisted reproduction at the Department of Reproductive Medicine and Gynaecological Endocrinology, University Medical Centre Maribor, Slovenia between 2018 and 2020.

The inclusion criteria were as follows: age ≤ 42 years, regular menstrual cycle 24 – 36 days in length, no current hormonal treatment, controlled ovarian stimulation (COS), ovulation triggering or vaginal progesterone for luteal support at least two months prior to endometrial biopsy. The exclusion criteria were anovulatory menstrual cycles, polycystic ovary syndrome (PCOS), previous surgical treatment of endometriosis or uterine surgical procedures, sonographic evidence of fibroids, endometrial polyps, hydrosalpinges, and evidence of ovarian or deep infiltrating endometriosis (unless otherwise noted in Table 1). In our clinic, all women undergoing assisted reproductive techniques (ART) have a prior transvaginal ultrasound (TVUS) examination, typically performed in the proliferative phase of the menstrual cycle. Women with echographic evidence of adenomyosis were considered eligible for the study, and the control group was composed of women with normal uteri seeking ART due to male or tubal factors of infertility.

On the day of endometrial sampling, all women underwent TVUS performed by a single expert sonographer (level 3 according to European Federation of Societies for Ultrasound in Medicine and Biology). In all women, comprehensive 2-D and 3-D ultrasound using high-range equipment was performed with a 10 MHz transvaginal transducer (Voluson E8 Expert, GE Health care, Austria GmbH & Co OG, Zipf, Austria). Diagnostic criteria for adenomyosis were based on previously published criteria [23]. The diagnosis of adenomyosis was confirmed when one of the following sonographic criteria was met: asymmetrical myometrial thickening not caused by the presence of fibroids, linear endometrial striations, irregular endometrial-myometrial junction, parallel shadowing, or the presence of myometrial cysts or hyperechoic islands [23]. Adenomyosis was classified as mild by subjective assessment, but in general, it was assessed in line with previously described principles. This was when only focal areas of adenomyosis were seen or when adenomyosis was present only in the inner third of the myometrium [24].

Demographic and clinical characteristics of participants, including age, body mass index (BMI), endometrial thickness at the time of endometrial biopsy and the number of previous ART cycles, are presented as the median (range) and were compared between study groups using the nonparametric Mann–Whitney U-test in SPSS 25.0 software (IBM Corporation, Armonk, NY, USA). Statistical significance was set at p value < 0.05.

### Endometrial sample collection

Endometrial biopsy sampling was conducted in a natural menstrual cycle, and women were scheduled for cycle monitoring by urinary luteinizing hormone (LH) tests (Hangzhou AllTest Biotech Co., Ltd, Hangzhou, P.R. China). Women were scheduled for endometrial sampling conducted by the Pipelle endometrial suction curette (the Probet, Gynetics Medical Products N.V., Lommel, Belgium) in the expected WOI on the day between LH+7 to LH+9 after a participant’s LH surge determination (day LH+0). Retrieved endometrial samples were immediately placed in RNAlater solution (Thermo Fisher Scientific Baltics UAB, Vilnius, Lithuania), stored overnight at +4 °C and then transferred to –80 °C until RNA isolation was performed.

### Total RNA isolation and quality control

Total RNA was isolated using the miRNeasy Mini Kit (Qiagen GmbH, Hilden, Germany) according to the manufacturer’s instructions. Each whole-tissue endometrial sample was first disrupted with a Bullet Blender Storm Pro homogenizer (Next Advance, lnc., Troy, NY, USA) using 1 mm zirconium oxide beads in 700 µL of QIAzol Lysis Reagent from the miRNeasy Mini Kit. After 5 min of incubation at room temperature, 140 µL of chloroform was added to the homogenate, and the solution was shaken vigorously. The sample was then centrifuged at 12 000 rfc for 15 min at 4 °C. The upper aqueous phase (approximately 300 µL) was transferred to a new Eppendorf tube, and 1.5 volumes of ethanol were added. The samples were then pipetted to RNA binding miRNeasy Mini spin columns and washed using RWT Buffer and RPE Buffer solutions of the miRNeasy Mini Kit. Total RNA was eluted in 50 µL of RNase-free H_2_O.

The quantity and purity of each RNA sample were assessed with Synergy 2 spectrophotometric measurements (BioTek Instruments, Winooski, VT, USA). RNA integrity number (RIN) was estimated on the 2100 Bioanalyser system (Agilent Technologies, Waldbronn, Germany) using the RNA Nano 6000 Assay Kit (Agilent Technologies, Waldbronn, Germany). After passing those quality controls, each RNA sample was used for cDNA library construction and subsequent RNA-seq and for accurate endometrial dating of retrieved biopsies.

### Accurate endometrial dating

One part of each RNA sample was shipped on dry ice to the Competence Centre on Health Technologies, CCHT, Tartu, Estonia, where endometrial receptivity testing was performed using the beREADY® test [25] (https://beready.ccht.ee/). Endometrial dating was performed according to the established protocol using targeted allele counting by sequencing (TAC-seq) methodology [26] to explore the expression levels of 57 well-described endometrial receptivity genes [27]. The results of the beREADY® test were provided in five phases: “pre-receptive”, “early-receptive“, “receptive”, “late-receptive”, and “post-receptive”. The purpose of endometrial dating was to accurately classify the receptivity status of LH-timed biopsies to remove samples that could lead to possible biases in gene expression analysis associated with endometrial receptivity in adenomyosis.

### Library preparation and RNA-seq

Both lncRNA and mRNA 150 bp paired-end libraries were constructed and subsequently sequenced by Novogene Bioinformatics Technology Co., Ltd. (Hong Kong, China). Briefly, a total amount of 2 µg of RNA per sample was used for cDNA sequencing library preparation. Ribosomal RNA (rRNA) was removed using the Epicentre Ribo-zeroTM rRNA Removal Kit (Epicentre, Brooklyn, NY, USA), and the remaining RNA was used for library generation by the NEBNext® UltraTM Directional RNA Library Prep Kit for Illumina® (NEB, Ipswich, MA, USA). First, rRNA-depleted RNA samples were fragmented followed by first- and second-strand cDNA synthesis. The sequencing adaptors were ligated, and library fragments were purified to obtain cDNA fragments 150~200 bp in length. Polymerase chain reaction (PCR) amplification of size-selected, adaptor-ligated cDNA was performed using universal PCR primers and index primers. Index-coded samples were clustered by Illumina TruSeq PE Cluster Kit v3-cBot-Hs. Libraries were sequenced on an Illumina NovaSeq 6000 platform, which generated 150 bp paired-end reads.

### RNA-seq data alignment and identification of DEGs

Raw sequence reads were trimmed by Novogene in-house Perlscript to remove raw reads with adapter contamination and reads containing poly-N and low-quality reads. The RNA-seq data presented in this study are deposited in the Gene Expression Omnibus (GEO) database with accession number GSE185392. Provided raw fastq files were first evaluated with FastQC v.0.11.9 software (http://www.bioinformatics.babraham.ac.uk/projects/fastqc/) to obtain a quality profile of the reads.

The statistical environment R v.4.0.2 (R Core Team 2020, Vienna, Austria) and contributed packages from the R software repository Bioconductor (http://www.bioconductor.org/) were used for high-throughput sequence data analysis. Raw paired-end reads were aligned to the UCSC Homo sapiens hg19 reference genome using the Rsubread v.2.2.4 R package [28, 29]. Properly mapped reads were sorted in files with binary alignment/map (BAM) format. Mapped reads were counted and assigned to genomic features using featureCounts [30] with the requirement that both ends should be mapped. Counts per million (CPMs) were calculated using the edgeR v.3.30.3 R package [31]. Genes expressed at low levels were filtered out based on CPMs corresponding to read counts of 10, and retained genes were normalized using the trimmed mean of M values method (TMM) [32]. Subsequently, mean-variance modelling at the observational level transformation (VOOM) was applied [33]. Differential expression analysis of the adenomyosis group relative to the control group was determined in two RNA-seq datasets using linear models and empirical Bayes implemented in the limma v.3.44.3 R package [34]. RNA-seq datasets were composed of libraries on the basis of the results of endometrial dating of corresponding samples. The first dataset contained all LH-timed samples, while the second dataset contained only samples dated to the receptive phase. Differential expression was considered for genes with a p value < 0.05 regardless of the adjusted p value obtained after multiple testing corrections.

### Integration of identified DEGs in the adenomyosis group with endometrial receptivity genes from the literature

Identified DEGs between adenomyosis cases and controls using samples dated to the receptive phase were applied for integrative bioinformatics analysis to provide robust candidate pathways associated with altered molecular background of endometrial receptivity in adenomyosis. DEGs were applied for enrichment reanalysis with lists of 42, 173 and 151 genes associated with endometrial receptivity in adenomyosis, endometriosis and healthy uterus, respectively, that were retrieved from the literature in our previous study [18]. Genes associated with endometriosis presented a model to study the impact of gynaecological pathology on endometrial molecular organization. Genes associated with a healthy uterus were used as a reference molecular background required for endometrial receptivity establishment. Two enrichment analyses were performed using two different gene lists associated with adenomyosis. The first adenomyosis gene list contained only 382 DEGs of the present sequencing experiment, while the second list combined 382 DEGs with 42 genes from the literature (in total, 424 genes). The first enrichment analysis was performed by integrating the adenomyosis gene list with 382 DEGs, the endometriosis list with 173 genes and the healthy uterus list with 151 genes. Second, enrichment analysis was performed using adenomyosis, healthy uterus and endometriosis lists with 424, 151 and 173 genes, respectively. The gene lists used are provided in Additional file 1.

### Functional enrichment analyses

DEGs (p<0.05) that were identified by transcriptomics data comparison of endometrial samples between adenomyosis cases and controls were subjected to functional enrichment analyses using ClueGO v.2.5.8 [35] and CluePedia 1.5.8 [36] apps of Cytoscape v.3.8.2 software [37]. The same bioinformatics tools were used for enrichment analyses employing integrated gene lists associated with adenomyosis, endometriosis and healthy uterus.

When analysing identified sets of DEGs associated with the present adenomyosis groups, up- and downregulated genes were separately uploaded as two clusters in the ClueGO app, which gave a unique colour marker to each gene set. When performing enrichment analyses of integrated gene lists associated with different gynaecological conditions, each gene list was uploaded as a cluster in the ClueGO app to distinguish study groups according to colour markers of the cluster.

Each enrichment analysis was applied by representative Gene Ontology Biological Process (GO_BP), Reactome Pathways and Reactome Reactions ontologies. Only enriched pathways (Reactome pathways/reactions and GO_BP terms) with corrected p values < 0.05 according to the Bonferroni step down test were considered. The identified pathways were sorted into groups based on their common biological role and associated genes (kappa score) and further projected into functionally organized networks. The size of nodes in the generated networks was correlated with the obtained p value. The pathway with the highest significant value was considered to be the leading term of a group and was therefore highlighted in the network by a large name label and a statistical summary. The CluePedia app was further applied to visualize shared initial genes within or between functional network groups. The proportion of visible genes mapped to each pathway was also determined. When more than 60% of mapped genes originated from one of the clusters, a pathway was shown in the network with the predefined colour of this cluster.

## Results

**Fig. 1:**
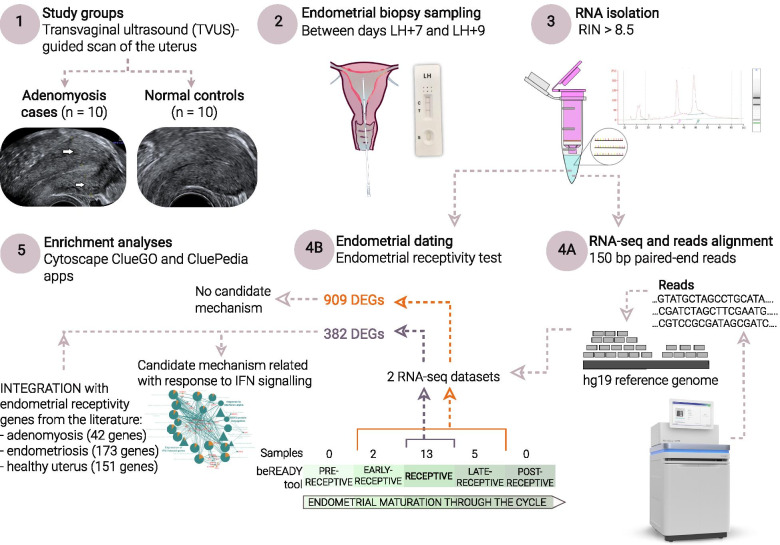
The overview of the present study. Only ovulatory women with regular menstrual cycles were included. Endometrial biopsies, 10 in the adenomyosis group and 10 the in control group, were conducted between 7 and 9 days post urinary LH peak corresponding to the expected WOI. Isolated RNA samples were used for RNA-seq using the Illumina NovaSeq 6000 platform and for accurate endometrial receptivity classification using the beREADY® molecular test. When analysing transcriptomics data of all 20 LH-timed biopsies, 909 DEGs (p < 0.05, nonsignificant after corrected p value) were associated with the adenomyosis group. Downstream functional enrichment analysis of these genes identified no strong candidate mechanisms associated with endometrial molecular biology. According to endometrial receptivity testing, 2 out of 20 samples were classified as early-receptive, 13 as receptive and 5 as late-receptive during the menstrual cycle. To prevent early- and late-secretory phases of the menstrual cycle on the transcriptomics analysis associated with endometrial receptivity, samples dated to the early- and late receptive phases were omitted from the RNA-seq dataset. The remaining transcriptomics data of 8 adenomyosis cases and 5 control samples dated to the receptive phase were reanalysed. The 382 identified DEGs (p <0.05, nonsignificant after corrected p value) in the adenomyosis group were further enriched in more robust candidate pathways, including “Expression of IFN-induced genes”, “Response to interferon-alpha” and “ISG15-protein conjugation”. The 382 identified DEGs were further integrated with 42, 173 and 151 genes from the literature associated with endometrial receptivity in adenomyosis, endometriosis and healthy uterus, respectively, to propose a molecular background of endometrial receptivity under adenomyosis. Abbreviations: DEGs = differentially expressed genes; LH = luteinizing hormone; RIN = RNA integrity number; WOI = window of implantation. The images of the NovaSeq 6000 Sequencing System and Gynetics suction curette were obtained from official pages Illumina.com and gyneatic.com, respectively

An overview of the study is outlined in Fig. 1.

### Participant characteristics

The demographic and clinical characteristics of the study cohorts are summarized in Table 1.

**Table 1:** Adenomyosis and control group characteristics

Characteristic	Adenomyosis group (N = 10)	Control group (N = 10)	p value
Age (years)	35 (30–39)	34.5 (30–42)	0.621
BMI (kg/m2)	27.2 (17.8–34.6)	21 (17.3–30.1)	0.112
Endometrial thickness (mm)	7.1 (4.6–11.2)	8 (6.2–10.1)	0.082
Number of performed ART cycles	2 (1–4)	4 (1–6)	0.353
Women sterility status:			
Primary sterility (nulligravid)	6	5	
Secondary sterility (gravida or parous)	4	5	
Factor of infertility:			
Male	5	8	
Tubal	2	1	
History of endometriosis	1	0	
Idiopathic infertility	2	1	

The median (range) is indicated for age, BMI, endometrial thickness and number of performed ART cycles (in vitro fertilization (IVF) and/or intracellular sperm injection (ICSI) treatments). P values are based on Mann–Whitney U test. Primary sterility refers to women who have never been pregnant (nulligravid), and secondary sterility refers to women who have already achieved pregnancy (gravida) or delivery (parous). Abbreviations: BMI = body mass index; ART = assisted reproductive technique

### Total RNA quality

Total RNA was isolated from 20 endometrial samples, 10 from the adenomyosis group and 10 from the control group. The A260/A280 ratios and RIN values of all RNA samples were above 2.0 and >8.5, respectively, and were further used for endometrial dating and RNA-seq.

### Endometrial receptivity classification of LH-timed biopsies

The results of endometrial receptivity testing performed on each endometrial RNA sample are provided in Table 2. According to the test, 13 out of 20 samples were classified in the receptive phase (8 adenomyosis cases and 5 controls), 2 samples in the early receptive phase, 5 samples in the late receptive phase and zero samples in the pre- or postreceptive phases.

**Table 2:** Characteristics of endometrial RNA samples used in the study

Sample ID	Day of biopsy sampling	Endometrial dating by the beREADY® test	Number of mapped RNA-seq reads	Proportion of mapped RNA-seq reads	Library size after normalization
A10	LH+8	receptive	70,040,785	97.97%	25,194,593
A12	LH+7	receptive	71,578,907	98.25%	24,095,230
A18	LH+8	receptive	59,462,597	97.85%	19,682,295
A20	LH+7	receptive	73,911,463	97.82%	26,033,066
A21	LH+8	late-receptive	66,603,640	98.34%	24,868,485
A29	LH+7	receptive	52,378,707	98.78%	19,079,266
A31	LH+7	receptive	71,617,925	98.78%	23,269,754
A3	LH+7	receptive	60,702,730	98.69%	19,575,933
A5	LH+7	receptive	63,795,289	98.02%	21,175,249
A9	LH+7	early-receptive	64,755,616	98.12%	26,091,668
K11	LH+9	receptive	60,841,108	98.02%	22,021,130
K15	LH+8	receptive	64,147,880	98.25%	23,535,991
K17	LH+8	late-receptive	64,945,168	97.84%	21,964,495
K22	LH+9	late-receptive	66,813,165	97.91%	22,118,116
K23	LH+8	late-receptive	50,492,938	98.76%	16,086,563
K24	LH+7	receptive	58,204,527	98.72%	17,532,809
K26	LH+9	late-receptive	55,574,782	98.87%	19,198,159
K27	LH+9	receptive	64,512,592	98.81%	19,919,022
K28	LH+7	early-receptive	57,037,427	98.63%	18,335,825
K8	LH+7	receptive	66,124,560	98.08%	25360,931

The timing of endometrial biopsy and measured receptivity status are provided for each sample, followed by a summary of mapped RNA-seq reads and library size after filtering for low gene expression. Abbreviations “A” refer to adenomyosis and “K” to control samples. Timing of biopsy refers to the day after luteinizing hormone (LH) peak determination (LH+0) by urinary LH test.

### RNA-seq outcome parameters

RNA-seq of endometrial samples utilizing the Illumina NovaSeq 6000 platform generated between 53,026,608 and 75,561,205 reads per sample, with an average of 64,266,576 reads. Quality analysis of raw RNA-seq reads by FastQC revealed that each fastq file contained reads 150 base pairs (bp) in length with a mean per base sequence quality score (Phred score) of 36 and thus each file was considered for downstream bioinformatics analysis. Table 2 summarizes the number and proportion of mapped raw reads to the hg9 reference genome for each sample and obtained library sizes after filtering low-expression genes.

### Identified DEGs associated with adenomyosis group

Differential expression analyses were conducted using two RNA-seq datasets constructed of samples according to the results of endometrial receptivity testing. The first RNA-seq dataset was composed of all 20 samples: 13 receptive, 2 early- and 5 late-receptive samples. The second RNA-seq dataset was composed of 13 receptive samples only, while 2 early- and 5 late-receptive samples were omitted to exclude the influence of early- and late-secretory phases of the menstrual cycle on endometrial transcriptomic analysis associated with endometrial receptivity.

Transciptomics data comparison of 10 adenomyosis and 10 control samples resulted in 909 DEGs (p<0.05) associated with the adenomyosis group (the entire list of 909 DEGs is presented in Additional file 2). According to the HUGO Gene Nomenclature Committee (HGNC) (version updated March 23, 2021) nomenclature system (https://www.genenames.org/), different locus types were identified, including 829 protein-coding genes (mRNAs), 27 long noncoding RNAs (lncRNAs), 5 microRNAs (miRNAs), 5 small nucleolar RNAs, 28 pseudogenes, 1 complex locus constituent and 14 loci that were not mapped in the HGNC database. Among 909 DEGs, 487 genes (452 mRNAs, 11 lncRNAs and remaining other loci types) were upregulated, and 422 genes (376 mRNAs, 16 lncRNAs and remaining other loci types) were downregulated. However, the fold change (FC) of expression levels between study groups was nonsignificant after the application of multiple comparison correction.

Transcriptomics data comparison of 8 adenomyosis cases and 5 control endometrial samples with confirmed receptive phase provided 382 DEGs (p < 0.05) associated with the adenomyosis group (the entire list of 382 DEGs is presented in Additional file 3). According to the HGNC nomenclature system, 323 loci were mRNAs, 23 lncRNAs, 21 pseudogenes, 4 miRNAs, 1 complex locus constituent, 1 T cell receptor gene and 9 uncharacterized. Among 382 DEGs, there were 166 upregulated (137 mRNAs, 14 lncRNAs and remaining other loci types) and 216 downregulated (186 mRNAs, 9 lncRNAs and remaining other loci types) genes. However, there were no significant DEGs between the study groups according to the adjusted p value. Among 382 DEGs in the adenomyosis group, up to the top 10 up- and downregulated mRNAs and lncRNAs with the highest logFC values of expression levels are presented in Tables 3 and 4, respectively.

**Table 3:** Top upregulated mRNAs and lncRNAs

ENTREZ ID	HGNC symbol	Long name	Locus type	logFC	p value
259289	*TAS2R43*	taste 2 receptor member 43	mRNA	0.9484	0.0215
4250	*SCGB2A2*	secretoglobin family 2 A member 2	mRNA	0.9244	0.0150
1747	*DLX3*	distal-less homeobox 3	mRNA	0.9212	0.0247
54959	*ODAM*	odontogenic, ameloblast associated	mRNA	0.9184	0.0460
353091	*RAET1G*	retinoic acid early transcript 1G	mRNA	0.8803	0.0346
84072	*HORMAD1*	HORMA domain containing 1	mRNA	0.8581	0.0031
563	*AZGP1*	alpha-2-glycoprotein 1, zinc-binding	mRNA	0.8259	0.0463
100507436	*MICA*	MHC class I polypeptide-related sequence A	mRNA	0.8127	0.0203
7348	*UPK1B*	uroplakin 1B	mRNA	0.8086	0.0224
158131	*OR1Q1*	olfactory receptor family 1 subfamily Q member 1	mRNA	0.8077	0.0466
100505967	*LINC00645*	long intergenic non-protein coding RNA 645	lncRNA	2.9900	0.0056
100130231	*LINC00861*	long intergenic non-protein coding RNA 861	lncRNA	2.0003	0.0177
654412	*FAM138B*	family with sequence similarity 138 member B	lncRNA	1.8938	0.0452
100505921	*GLCCI1-DT*	GLCCI1 divergent transcript	lncRNA	1.8543	0.0323
100506334	*LINC00649*	long intergenic non-protein coding RNA 649	lncRNA	1.7910	0.0314
284578	*MFSD4A-AS1*	MFSD4A antisense RNA 1	lncRNA	1.6098	0.0022
283876	*LINC00921*	long intergenic non-protein coding RNA 921	lncRNA	1.3284	0.0443
100505625	*LINC02102*	long intergenic non-protein coding RNA 2102	lncRNA	1.3182	0.0300
100507398	*INTS6-AS1*	INTS6 antisense RNA 1	lncRNA	1.2787	0.0023
93653	*ST7-AS1*	ST7 antisense RNA 1	lncRNA	1.2489	0.0120

DEGs were insignificant after multiple testing correction of the p value. Abbreviation “FC” refers to fold change of expression levels

**Table 4:** Top downregulated mRNAs and lncRNAs

ENTREZ ID	HGNC symbol	Long name	Locus type	logFC	p value
169693	*TMEM252*	transmembrane protein 252	mRNA	-2.1611	0.0331
5655	*KLK10*	kallikrein related peptidase 10	mRNA	-1.7433	0.0010
5803	*PTPRZ1*	protein tyrosine phosphatase receptor type Z1	mRNA	-1.7022	0.0120
727897	*MUC5B*	mucin 5B, oligomeric mucus/gel-forming	mRNA	-1.6678	0.0421
79937	*CNTNAP3*	contactin associated protein like 3	mRNA	-1.5039	0.0096
10752	*CHL1*	cell adhesion molecule L1 like	mRNA	-1.5027	0.0391
7103	*TSPAN8*	tetraspanin 8	mRNA	-1.4535	0.0070
9723	*SEMA3E*	semaphorin 3E	mRNA	-1.4216	0.0001
10964	*IFI44 L*	interferon induced protein 44 like	mRNA	-1.3304	0.0416
5340	*PLG*	plasminogen	mRNA	-1.3219	0.0499
145837	*DRAIC*	downregulated RNA in cancer, inhibitor of cell invasion and migration	lncRNA	-1.9848	0.0392
100131825	*CADM3-AS1*	CADM3 antisense RNA 1	lncRNA	-1.6197	0.0298
100506674	*MRPS30-DT*	MRPS30 divergent transcript	lncRNA	-1.2230	0.0049
641364	*SLC7A11-AS1*	SLC7A11 antisense RNA 1	lncRNA	-0.7862	0.0302
100506305	*LINC00958*	long intergenic non-protein coding RNA 958	lncRNA	-0.6847	0.0448
100289410	*MCF2 L-AS1*	MCF2 L antisense RNA 1	lncRNA	-0.6591	0.0414
386597	*RNF144A-AS1*	RNF144A antisense RNA 1	lncRNA	-0.6095	0.0177
144481	*SOCS2-AS1*	SOCS2 antisense RNA 1	lncRNA	-0.4608	0.0205
100134229	*KDM7A-DT*	KDM7A divergent transcript	lncRNA	-0.4579	0.0344

DEGs were insignificant after multiple testing correction of the p value. Abbreviation “FC” refers to fold change of expression levels

### Enriched pathways associated with identified DEGs

**Fig. 2:**
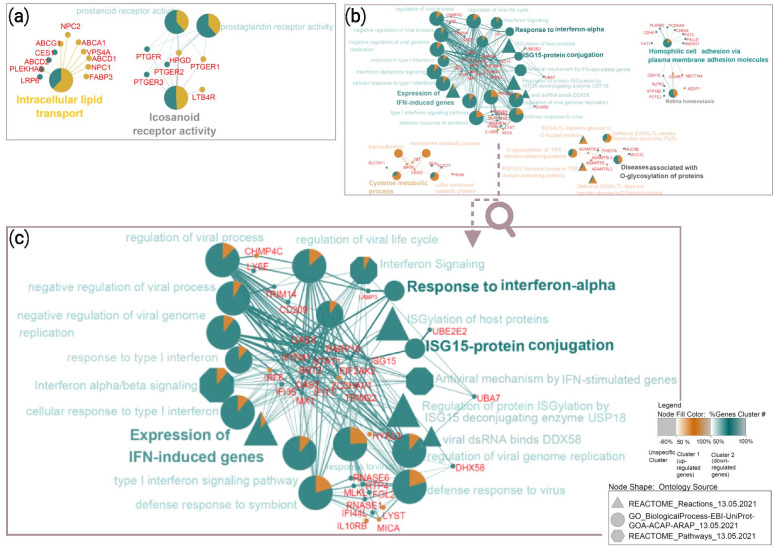
Networks of enriched pathways and mapped genes associated with DEGs in the adenomyosis group. (**a**) Sorted 4 pathways in 2 network groups obtained by the enrichment analysis of 909 DEGs associated with adenomyosis group after comparing receptive, early- and late-receptive case and control samples; (**b**) Sorted 33 enriched pathways within 7 network groups identified from 382 DEGs associated with adenomyosis group after comparing only case and control samples dated to the receptive phase; (**c**) The enlargement of the connected network groups “Expression of IFN-induced genes”, “Response to interferon-alpha” and “ISG15-protein conjugation” presenting candidate pathways for future studies associated with altered endometrial receptivity in adenomyosis. Each set of DEGs was uploaded in the Cytoscape ClueGO app as two separate clusters, where upregulated genes were marked with violet and downregulated genes with green colour. Shape of nodes in networks attributed to ontology sources that were applied for enrichment analysis. Enriched pathways were sorted into network groups based on their common biological role

Functional enrichment analysis of 909 DEGs associated with the adenomyosis group that were obtained from transcriptomics comparison of all 20 endometrial samples provided only 4 enriched GO terms sorted within 2 functionally organized network groups: “Intracellular lipid transport” (11 mapped genes, corrected p value 2.15 × 10^-5^) and “Icosanoid receptor activity” (6 mapped genes, corrected p value 2.02 × 10^-5^). Sorted pathways with associated genes in networks are presented in Fig. 2a. The results of the enrichment analysis are summarized in Additional file 4.

Functional enrichment analysis of 382 DEGs associated with the adenomyosis group that were obtained by transcriptomics analysis of endometrial samples in the receptive phase resulted in 33 enriched pathways, including 20 GO_BP terms, 6 Reactome pathways and 7 Reactome reactions. They were sorted into 7 network groups to remove redundancy, which is visualized in Fig. 2b. The highest proportion of enriched pathways was related to mechanisms of response to interferon (IFN) signalling, in particular antiviral response (presented in higher resolution in Fig. 2c). Most of the downregulated genes were mapped in the following network groups: “Expression of IFN-induced genes” (*BST2*, *IFI35*, *IFIT1*, *IFITM1*, *ISG15*, *MX1*, *OAS2*, *OAS3* and *STAT1* were down- and *IRF6* was upregulated, corrected p value 2.08 × 10^-6^), “Response to interferon-alpha” (*BST2*, *EIF2AK2*, *IFITM1*, and *LAMP3*, corrected p value 8.75 × 10^-4^), “ISG15-protein conjugation” (*ISG15*, *UBA7* and *UBE2E2*, corrected p value 3.02 × 10^-5^) and “Homophilic cell adhesion via plasma membrane adhesion molecules” (*AMIGO1*, *CDH15*, *CDH24*, *CDH6*, *FAT1*, *FAT2*, *PALLD*, *PCDHA9* and *PLXNB3* were down-, while *CDHR1* and *NECTIN4* were upregulated, corrected p value 5.75 × 10^-4^). Upregulated genes were mapped in the specific network group “Cysteine metabolic process” (*MPST*, *TST* and *VSIG2* were up- and *SLC7A11* was downregulated, corrected p value 5.62 × 10^-4^). Nonspecific network groups characterized by equal proportions of mapped up- and downregulated genes were “Diseases associated with O-glycosylation of proteins” (*ADAMTS17*, *ADAMTS5* and *ADAMTSL2* were up-, while *ADAMTSL1*, *MUC13*, *MUC5B* and *THSD7A* were downregulated, corrected p value 4.48 × 10^-4^) and “Retina homeostasis” (*AZGP1*, *CDHR1* and *NECTIN4* were up-, while *ALPK3*, *ATP1B2*, *CDH15* and *POTEJ* were downregulated, corrected p value 6.42 × 10^-4^). The 33 identified enriched pathways are summarized in Additional file 5.

### Enriched pathways obtained by integration of identified DEGs and endometrial receptivity genes from the literature

Only a set of 382 DEGs associated with the adenomyosis group that were identified by transcriptomics data comparison of adenomyosis case and control samples dated to the receptive phase were used for integrative enrichment analyses with endometrial receptivity genes from the literature.

**Fig. 3:**
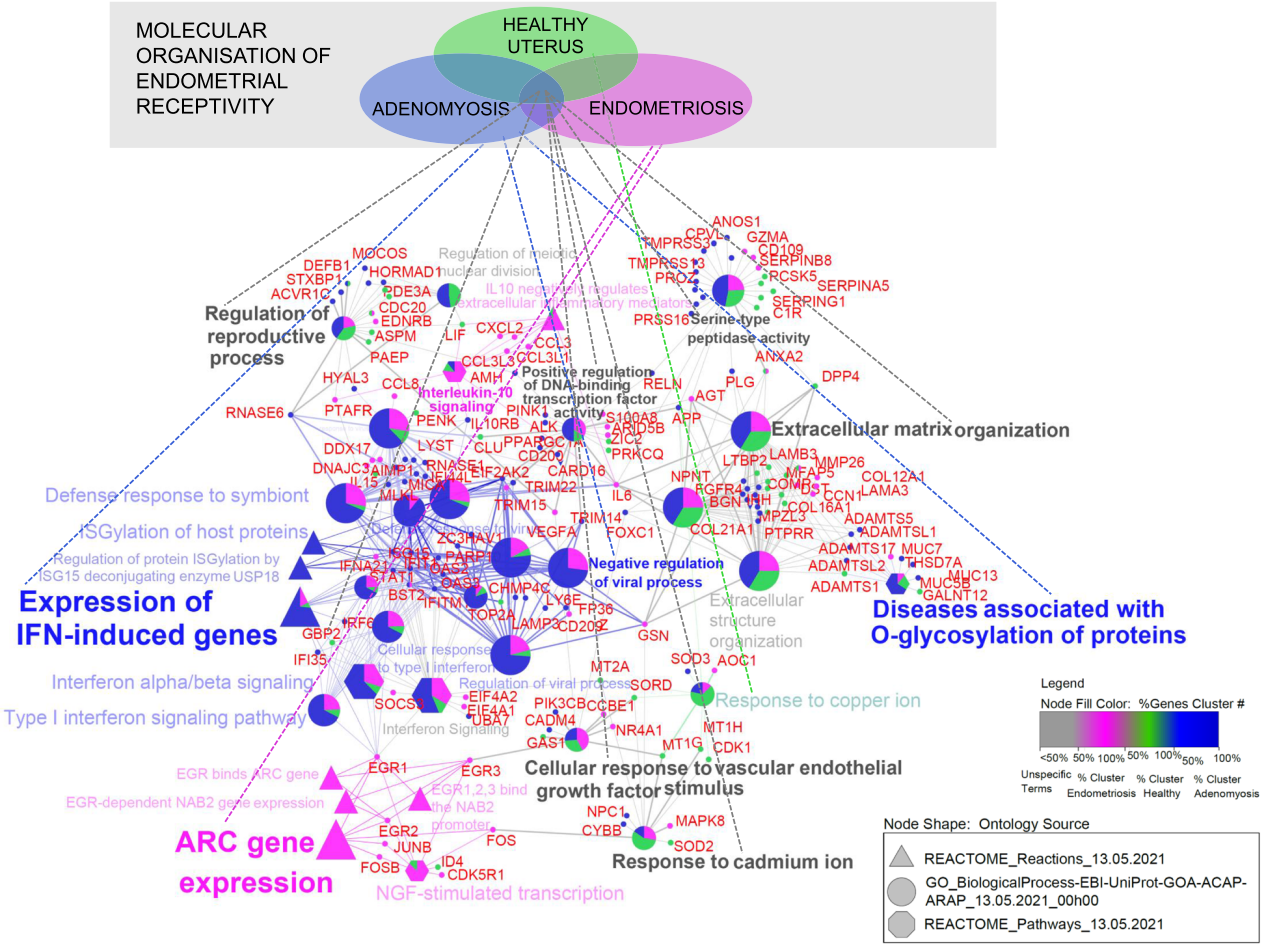
Integration of 382 adenomyosis-associated DEGs with endometrial receptivity genes associated with endometriosis and healthy uterus. In total, 40 pathways sorted into 11 network groups were obtained after enrichment analysis of integrated gene lists. The adenomyosis gene list (blue colour) included 382 DEGs associated with the adenomyosis group of the present RNA-seq analysis. The healthy uterus list (green colour) and endometriosis list (pink colour) contained 151 and 173 genes, respectively, which were associated with endometrial receptivity in the literature

Integration of lists with 382, 151 and 173 genes associated with adenomyosis, healthy uterus and endometriosis, respectively, provided 40 enriched pathways sorted in 11 network groups, which are presented in Fig. 3. According to the generated network, unique fingerprints of gynaecological pathologies on endometrial signatures were observed. The identified “Expression of IFN-induced genes”, “Negative regulation of viral process” and “Diseases associated with O-glycosylation of proteins” network groups were specific for the adenomyosis gene list, while “Interleukin-10 signalling” and “ARC gene expression” were specific for the endometriosis gene list. In addition, nonspecific network groups, characterized by mapped genes originating from all 3 lists associated with gynaecological conditions, were identified, including “Extracellular matrix organization”, “Serine-type peptidase activity”, “Positive regulation of DNA-binding transcription factor activity”, “Cellular response to vascular endothelial growth factor stimulus”, “Response to cadmium ion” and “Regulation of reproductive process”. This could indicate the interference of adenomyosis and endometriosis with molecular mechanisms required for normal endometrial receptivity. The 40 identified enriched pathways are summarized in Additional file 6.

**Fig. 4:**
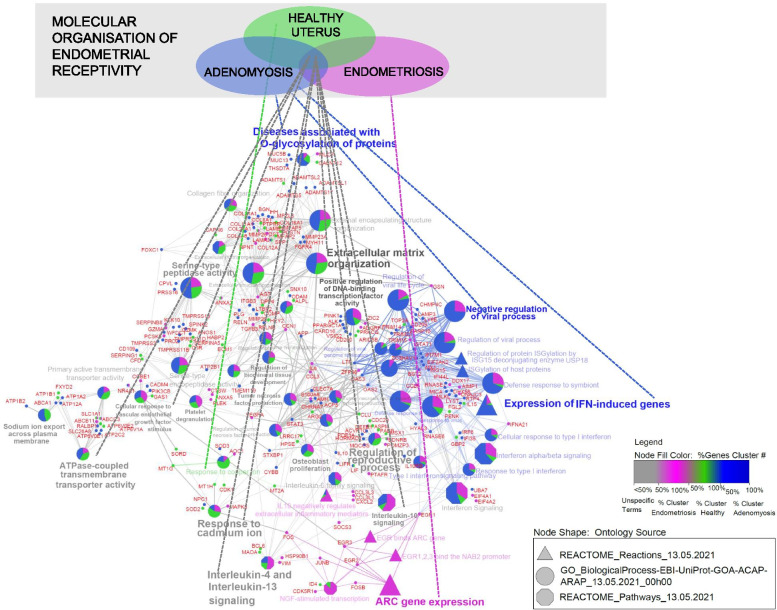
Integration of 424 adenomyosis-associated DEGs with endometrial receptivity genes associated with endometriosis and healthy uterus. In total, 57 enriched pathways sorted into 18 network groups were obtained after integration of adenomyosis list with 424 genes (382 DEGs of the present experiment and 42 genes from the literature), (blue colour), endometriosis list with 173 genes (pink colour) and healthy uterus list with 151 genes (green colour)

Integration of lists with 424 (382 DEGs of the present sequencing experiment and 42 genes from the literature), 151 and 173 genes associated with adenomyosis, healthy uterus and endometriosis, respectively, provided 57 enriched pathways sorted in 18 network groups, which are presented in Fig. 4. Similar results were retrieved with the integration of the adenomyosis gene list with 382 DEGs alone. However, some additional nonspecific network groups were identified, including “Interleukin-4 and Interleukin-13 signalling”, “Tumour necrosis factor production” and “Sodium ion export across plasma membrane”. The 57 identified enriched pathways are summarized in Additional file 7.

## Discussion

Altered endometrial molecular mechanisms obstructing successful embryo implantation in women with adenomyosis are poorly understood. The focus of the present transcriptomics analysis was to apply two novel molecular approaches to identify gene expression differences in LH-timed endometrial samples between women with and without adenomyosis: genome-wide profiling using RNA-seq and accurate classification of endometrial receptivity as assessed by the molecular tool beREADY®, measured from the same biopsy. Lists of DEGs associated with the adenomyosis group that were identified by analysing RNA-seq datasets in the setting of the endometrial dating results were applied for enrichment pathway analyses to predict their role in the context of endometrial molecular organization. In addition, a set of 382 DEGs obtained after transcriptomics data comparison of confirmed receptive samples was used for further bioinformatics analysis. They were integrated with the most extensive set of genes from the literature associated with endometrial receptivity in healthy uterus, endometriosis (model disease to study persistence of gynaecological pathology on endometrial molecular organization) and adenomyosis to predict mechanisms in which adenomyosis mediates an effect on endometrial receptivity.

Recently, Devesa-Peiro et al. [38] compared available transcriptomics data and observed a greater effect of changing phases of the menstrual cycle on the endometrial transcriptome signature than the persistence of endometrial pathologies. Accurate dating of collected biopsies was highlighted as an important procedure when identifying endometrial biomarkers associated with uterine abnormalities [38]. In addition, displacement of the temporal appearance of the WOI has been observed in some women [39, 40], meaning that the WOI could appear earlier or later in the luteal phase, as it is generally assumed that it is constant in all women [39, 41]. In view of these data, we utilized the novel molecular beREADY® tool [25], which reliably determines endometrial dating on a transcriptomics platform, and machine-learning algorithms to assure homozygosity of LH-timed biopsies in the present study groups. Considering the results of endometrial receptivity testing, we excluded early- and late-receptive samples from the RNA-seq dataset to prevent the impact of early- and late-secretory phases associated with physiological advancement of endometrial maturation through the menstrual cycle, which could bias transcriptomics analysis associated with endometrial receptivity in adenomyosis. In that way, we identified 382 DEGs that we believe more accurately represent the effect of adenomyosis on the gene expression signature of endometrial receptivity compared to 909 DEGs associated with the adenomyosis group, which were identified by comparing transcriptomics data of samples derived from receptive, early- and late-receptive phases.

Enrichment analysis using 382 DEGs also provided a higher number of pathways tightly sorted in connected network groups compared to analysis of 909 DEGs, which were also more meaningful to relate with endometrial molecular biology (Fig. 2b). Namely, according to the results of the enrichment analysis of 382 DEGs, “Expression of IFN-induced genes”, “Response to interferon-alpha” and “ISG15-protein conjugation” were sorted as connected processes (Fig. 2c). Popovici et al. [42] associated increased expression levels of genes encoding chemotactic factors, inflammatory cytokines (including type I IFN-alpha/beta) and apoptosis-inducing agents with a role in the recruitment of lymphocytes and macrophages in human endometrial decidua [42]. IFNs, as reviewed by De Veer et al. [43], are a family of multifunctional cytokines that activate the expression of many genes with antiviral, antiproliferative or immunosuppressive effects. The signal transduction pathway of IFNs is initiated upon IFN binding to specific cell surface receptors. Downstream formed complexes of phosphorylated proteins and transcription factors bind to IFN-stimulated response elements (ISREs) at the promotor region of IFN-stimulated genes (ISGs) and initiate their transcription. There are more than 300 ISGs [43]. The ubiquitin-like protein ISG15 is a posttranscriptional modifier that can be in a process termed ISGylation covalently linked to hundreds of proteins. The role of ISG15 has been associated with cellular processes such as protein translation, cytoskeleton dynamics, exosome secretion, autophagy, genome stability and cancer; therefore, it presents a potential target for therapeutic strategies [44]. ISG15 can exert functions as an intracellular and secreted protein. Intracellular expression of ISG15, which is dependent on type I IFN-alpha/beta signalling, characterizes innate immune responses to viral and microbial pathogens. Its extracellular signalling can elicit secretion of cytokine type II IFN-gamma from lymphocytes [45]. Studies in mice suggested that ISG15 plays a role in the recruitment of uterine natural killer (uNK) cells during early gestation, where it is responsible for remodelling of spiral arteries to ensure a normal blood supply to the foetus and placenta throughout pregnancy [46]. The identified enriched pathways related to the response to IFN signalling could indicate altered immune factors that have been associated with adenomyosis. Tremellen and Russell [47] associated an increased density of uNK cells and macrophages in the functional layer of late-secretory endometrium in women with severe adenomyosis experiencing implantation failures with a hostile immune environment that might interfere with successful embryo implantation [47]. In addition, Sotnikova et al. [48] reported higher levels of secreted proinflammatory cytokines (IFN-gamma, IFN-alpha, tumour necrosis factor (TNF)-alpha and interleukin (IL)-1 beta) in supernatant samples of cultured mononuclear cells obtained from late-secretory endometrium of women with adenomyosis when compared with healthy controls [48]. Another interesting enriched pathway from the 382 DEGs was related to cellular adhesion, whose importance in the process of embryo implantation has been described elsewhere [49].

The 382 Identified DEGs were also applied for the integration approach to repeat our previous enrichment pathway analysis [18] oriented to detect candidate pathways of affected endometrial receptivity in adenomyosis. Integrative enrichment analysis using the adenomyosis gene list with 382 DEGs only provided candidate pathways associated with endometrial receptivity establishment (e.g., “Extracellular matrix organization” “Cellular response to vascular endothelial growth factor stimulus” and “Regulation of reproductive process”) that could be dysregulated in adenomyosis as well as in endometriosis, which is in agreement with the literature [17, 50–54]. The identified specific network group “Expression of IFN-induced genes” persisted as a unique effect of adenomyosis on endometrial molecular background after enrichment analysis using integrated gene lists. Enriched pathways related to activity-regulated cytoskeletal (ARC) gene expression were specific to the endometriosis gene list, which was used as a model to study the effect of endometrial-associated disorders. ARC is an immediate early gene involved in signal transduction. Its transcription is induced by various signalling cascades, including mitogen-activated protein kinases (MAPKs) and extracellular signal-regulated kinases (ERKs) [55], which have already been associated with endometrial receptivity defects in endometriosis [56]. Integrative enrichment analysis using the adenomyosis gene list with 424 genes provided additional candidate pathways to be associated with altered cytokine responses in adenomyosis and endometriosis, including “interleukin-4 and interleukin-13 signalling”, which was also identified in our previous study [18] and could be attributable to the Reactome pathway database being used as an ontology source in both studies, “regulation of TNF superfamily cytokine production” and “interleukin-10 signalling”. Altered expression levels of some cytokines in the endometrium during WOI have been observed in women with adenomyosis after COS [57] and in women with endometriosis [58, 59]. Decidualization of endometrial stromal cells is characterized by a changing endometrial inflammatory environment shown as a transition from a proinflammatory to an anti-inflammatory response [60, 61]. This transition has been associated with balancing endometrial receptivity versus selectively accepting only high-quality embryos [61]. Dysregulated balance has been associated with the implantation of poor-quality embryos leading to miscarriage [62]. It could be that enriched pathways associated with the expression of IFN-induced genes indicate dysregulated endometrial selectively, which may explain the observed higher incidence of early pregnancy loss in women with adenomyosis [6, 20]. However, further studies are needed to verify this hypothesis. The identification of robust pathways could lead to the extension of current gene sets for endometrial receptivity examination presented in a growing number of commercial molecular tests [25, 63–65] that would be specific for women with adenomyosis. Accurate endometrial receptivity examination in this group of infertile patients could better verify whether endometrial-associated factor is a source of recurrent implantation failures that prolong infertility treatments [66]. Furthermore, endometrial changes in women with adenomyosis could provide not only the relationship between pathophysiological mechanisms of adenomyosis development [17, 67, 68] but also the pathogenesis of the malignant transformation [69]. Recently, it was reported that endometrial carcinoma could co-exist or arise from adenomyosis which may be important factor in survival outcomes of the patient [70, 71].

A limitation of our study is the relatively small sample size, which prevents definitive conclusions regarding the impact of adenomyosis on the endometrial transcriptome [72]. The results could also differ if the control group is composed of women with proven fertility. Another limitation of the present study is that the diagnosis of adenomyosis could only be made noninvasively by imaging, since definitive histopathological diagnosis can only be made after hysterectomy. In genome-wide studies focusing on pathophysiological aspects of adenomyosis, the diagnosis can be based on histological examination of specimens after hysterectomy [17, 67, 68]. However, this is only possible retrospectively and is irrelevant in women who wish to preserve their fertility. In fertility-oriented transcriptomics studies [20] or studies including endometriosis [73], a diagnosis of adenomyosis was noninvasive. The diagnosis of adenomyosis by ultrasound is challenging, and there are no uniform ultrasonographic criteria for the diagnosis [74]. In the present study, TVUS of the uterus and pelvic cavity was performed by an experienced sonographer prior to each endometrial biopsy to confirm sonographic evidence of adenomyosis and to exclude other pelvic pathologies.

## Conclusions

In this study, we focused on the molecular background of infertility-related adenomyosis based on our research and the available literature. We applied accurate endometrial receptivity classification of retrieved endometrial samples LH-timed to the expected WOI to avoid menstrual cycle bias in downstream transcriptomics analysis. The 382 DEGs identified in the adenomyosis group using the RNA-seq dataset of only confirmed receptive endometrial samples resulted in 33 enriched pathways further projected in the network from which “Expression of IFN-induced genes”, “Response to interferon-alpha” and “ISG15-protein conjugation” were highlighted as connected processes. Additional integration of 382 DEGs with candidate genes associated with endometrial receptivity in healthy uterus, endometriosis and adenomyosis based on a literature review revealed that cytokine signalling impairments in endometrial pathologies could interfere with mechanisms of endometrial receptivity. According to our results, an altered response to IFN signalling is suggested as a candidate mechanism of impaired uterine receptivity in adenomyosis that needs to be further studied in a larger sample size.

## Supplementary Information

**Additional file 1.**

**Additional file 2.**

**Additional file 3.**

**Additional file 4.**

**Additional file 5.**

**Additional file 6.**

**Additional file 7.**

## Data Availability

All data generated and analysed during this study are included in this article [and its supplementary information file]. The RNA-seq data presented in this study are deposited in the GEO database with accession number GSE185392.

## Abbreviations

ART: Assisted reproductive technique
DEG: Differentially expressed gene
GO: Gene ontology
IFN: Interferon
LH: Luteinizing hormone
RNA-seq: RNA sequencing
TVUS: Transvaginal ultrasound
WOI: Window of implantation
